# Optimization of the Ugi Reaction Using Parallel Synthesis and Automated Liquid Handling

**DOI:** 10.3791/942

**Published:** 2008-11-11

**Authors:** Jean-Claude Bradley, Khalid Baig Mirza, Tom Osborne, Antony Wiliams, Kevin Owens

**Affiliations:** Department of Chemistry, Drexel University; Mettler-Toledo; Chemspider

## Abstract

The optimization of a Ugi reaction involving the mixing of furfurylamine, benzaldehyde, boc-glycine and t-butylisocyanide is described.  Triplicate runs of 48 parallel experiments are reported, varying concentration, solvent and the excess of some of the reagents.  The isolation of the product was achieved by a simple filtration and wash procedure.  The highest yield obtained was 66% from 0.4 M methanol with 1.2 eq. of imine. This is significantly above the 49% yield obtained from the initial reaction under equimolar concentration at 0.4 M in methanol.  Methanol solutions with reagent concentrations of 0.4M or 0.2M gave superior yields while all solvent systems at 0.07M performed poorly.  At 0.2M, methanol and ethanol/methanol (60/40) mixtures were statistically equally good while THF/methanol (60/40)  was poor and acetonitrile/methanol (60/40) was intermediate. Good reproducibility of the precipitate yields was obtained in these replicate experiments, allowing for subtle interaction effects to be positively identified.

**Figure:**
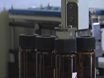


## Protocol

### Introduction

The Ugi reaction has proved to be a convenient way to quickly create diverse libraries of compounds (1-3). It involves the reaction of an amine, an aldehyde, a carboxylic acid and an isonitrile typically in methanol at room temperature. The Ugi reaction has often been used as a tool in the synthesis of pharmacologically active molecules and we have exploited it to quickly access compounds in the search for new anti-malarial agents (4). It has been observed that Ugi products sometimes precipitate in pure form from the reaction mixture (1,5). This is a very fortunate outcome since the reaction can then be easily scaled up without requiring costly purification procedures such as chromatography. It would be most beneficial to optimize the Ugi product yield as obtained directly from filtering the reaction mixture without further treatment. To this end we utilized a 48-slot Mettler-Toledo MiniBlock (6) equipped with filtration tubes. A Mettler-Toledo MiniMapper (7) automated liquid handler was used to deliver the reagents and solvent. The parameters of interest were the concentration, the solvent composition and the excess of some of the reagents.

### Experimental

The MiniMapper automated liquid handler was programmed to deliver liquids in the following sequence to empty filter tubes in a 48-position MiniBlock: additional solvent, furfurylamine (2M in methanol), benzaldehyde (2M in methanol), boc-glycine (2M in methanol) and t-butylisocyanide (2M in methanol). The default addition volume was 100 microliters. If a reagent is noted as being added in excess, 120 microliters were delivered. The MiniBlock was then placed on a shaker for 16 hours before being filtered using house vacuum. Two washes are performed by adding methanol (1 mL) to each tube followed by 15 min shaking before filtering off. The tubes were then dried under high vacuum in a dessicator for at least 30 min. The yield was calculated from the increase in weight of the filter tubes. The purity was assessed by H NMR for one sample from each solvent system and concentration. The reactions were run in triplicate (8-10) and the average yields are reported in Table 1.

### Characterization of the Ugi product

tert-butyl (2-{[2-(tert-butylamino)-2-oxo-1-phenylethyl](furan-2-ylmethyl)amino}-2-oxoethyl)carbamate : white solid; m.p.(11) 202-204 C; 1H NMR (12, spectrum) (500MHz,δppm, CDCl3) 1.33 (s, 9H), 1.45 (s, 9H), 4.21 (m, 2H), 4.49 (d, J=18Hz, 1H), 4.50 (d, J=18Hz, 1H), 5.47 (s, 1H), 5.60 (s, 1H), 5.62 (s, 1H), 5.89 (s, 1H), 6.10 (s, 1H), 7.19 (s, 1H), phenyl 7.21-7.37 (m, 5H); 13C NMR (13, spectrum) (500MHz, δppm, CDCl3) 28.3, 28.6, 42.3, 42.8, 51.7, 62.9, 79.5, 107.7, 110.4, 128.5, 128.7, 129.6, 134.7, 141.9, 149.8, 155.7, 168.4 170.2; IR (14, spectrum) (Ʋmax cm-1 ATR): 1645,1673,1699, 3331; FAB-HRMS (15, spectrum) (calculated for C24H33N3O5 m/z 444.2498 [M+H], obtained 444.2517.)

### Data Analysis

The precipitate yield data was analyzed using the single-factor or two-factor (with replicates) analysis of variance (ANOVA) tools available in Microsoft Excel. (16) For those variables found to be statistically significant in the ANOVA analysis, Fisher's Least Significant Difference (LSD) test was used to determine the specific experimental settings exhibiting significant differences in yield of the Ugi precipitate (17). All significance tests were performed at the 95% confidence level.

### Results and Discussion

****Figure 1 summarizes the effect of solvent composition on the yield of the Ugi precipitate. A single factor ANOVA indicates a significant difference in average yield for the four solvents studied at a reagent concentration of 0.2M. Fisher's LSD test indicates that the precipitate yields obtained in methanol and ethanol are not statistically different at the 95% confidence level (signified by the bar over the names of solvents in the figure); the yield of Ugi precipitate is significantly greater in ethanol and methanol than in acetonitrile, which in turn is significantly greater than that in THF.


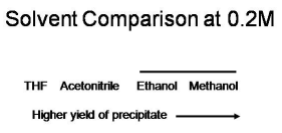


**Figure 1:** Summary of the effect of solvent composition on the yield of Ugi precipitate at a reagent concentration of 0.2M. The listed solvents include 40% methanol at this concentration.

Considering the effect of reagent concentration, the data in Table 1 show a precipitous drop in yield was found when going from 0.2M to 0.07M using methanol, ethanol and acetonitrile as the solvent; the yields were comparable in methanol at the 0.2M and 0.4M concentrations. Note that Ugi reactions are generally run in the 0.5-2M range (2). However, when using an automated liquid handler, it may be difficult to start with reagent solutions much more concentrated than 2M, thus placing a practical upper limit on the final concentration at about 0.5M. The results of this optimization study suggest caution in interpreting the success or failure of Ugi reactions below 0.2M, since non-linear effects are apparently at play.

Figure 2 summarizes the effect of solvent composition and reagent excess on the yield of Ugi precipitate at a reagent concentration of 0.2M. The two-factor ANOVA results indicate a statistically significant solvent and reagent excess effect. In addition, there is a statistically significant interaction between the two variables. Details of the interaction are uncovered in figure 2 which shows that the yield of precipitate is affected by the solvent used. Again note that the bars over the factors in the figure indicate the yields are not statistically different at the 95% confidence level. For both ethanol and methanol the best yields are obtained with the imine or isonitrile in excess, while for acetonitrile, significantly improved results are obtained with the amine, aldehyde or isonitrile in excess. As noted above in the discussion of the results in Table 1, the overall yield of precipitate is low for THF as the solvent, and only an excess of imine leads to a statistically significant improvement in precipitate yield. These different patterns of improved yield for the four solvents is what leads to the statistically significant interaction effect, and may be an indicator of different interaction of the Ugi product with the different solvents. Although the numerical results in Table 1 show the best yield was obtained in 0.4M methanol with 1.2 equivalents of excess imine, 0.2M acetonitrile/methanol and 0.2M ethanol/methanol mixtures also provided significant yields. Note particularly that all of the THF/methanol mixtures at 0.2M gave very poor results. The Ugi reaction has been successfully carried out in a variety of solvents, including THF (2). Note that the low yields here do not imply that the Ugi reaction did not take place in THF mixtures, only that a precipitate was not formed.


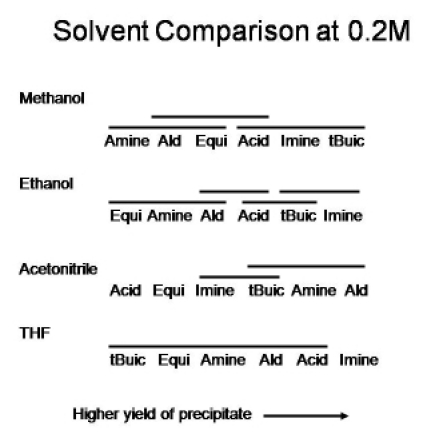


**Figure 2:** Summary of the effect of solvent composition and reagent excess on the yield of Ugi precipitate at a reagent concentration of 0.2M. The listed solvents include 40% methanol at this concentration.

Figure 3 summarizes the effect of reagent concentration and reagent excess on the yield of Ugi precipitate using methanol as the solvent. The two-factor ANOVA results indicate a statistically significant concentration and reagent excess effect. As described above, the yield of precipitate at reagent concentrations of of 0.2M and 0.4M are significantly greater than at 0.07M (but do not differ from each other). In addition, there is a statistically significant interaction between the two variables under study. Note that the pattern of the improvement in precipitate yield with identity of the excess reagent is generally the same at the higher reagent concentrations.


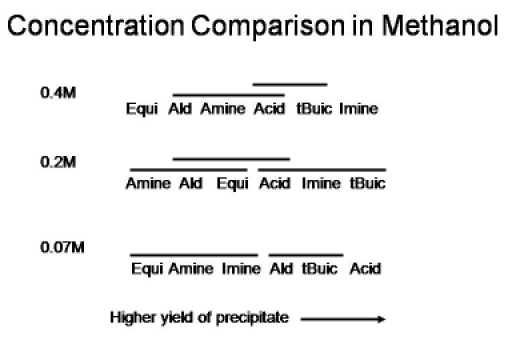


**Figure 3: **Summary of the effect of concentration and reagent excess on the yield of Ugi precipitate in methanol solvent.

Overall, the results described in figures 2 and 3 demonstrate that there were interactions between the solvent choice, reagent concentration and identity of the reagent in excess, but overall these were more subtle than the straight solvent and concentration effects. Others have found similar results when optimizing a Ugi reaction (18).

****

### Conclusion

The optimization experiment found the highest yield of the 48 runs to be 66% from 0.4 M methanol with 1.2 eq. of imine. This is significantly above the 49% yield obtained from the initial reaction under equimolar concentration at 0.4 M in methanol. Good reproducibility of the precipitate yields was obtained in these replicate experiments, allowing for subtle interaction effects to be positively identified.

## References

[B0] Marcaccini S, Torroba T (2007). The use of the Ugi four-component condensation. Nature Protocols.

[B1] Domling A, Ugi I (2000). Multicomponent reactions with isocyanides Angew. Chem. Int. Eng. Ed.

[B2] Domling A (2006). Recent Developments in Isocyanide Based Multi-Component Reactions in. Applied Chemistry, Chem. Rev..

[B3] Bradley J-C http://usefulchem.blogspot.com/2008/01/we-have-anti-malarial-activity.html.

[B4] Sanudo M, Marcaccini M, Basurto S, Torroba T (2006). Synthesis of 3-Hydroxy-6-oxo[1,2,4]triazin-1-yl Alaninamides, a New Class of Cyclic Dipeptidyl. Ureas J. Org. Chem..

[B5] Bradley J-C, Mirza KB UsefulChem EXP201 http://usefulchem.wikispaces.com/EXP201.

[B6] Bradley J-C, Mirza KB EXP202 http://usefulchem.wikispaces.com/EXP202.

[B7] Bradley J-C, Mirza KB EXP203 http://usefulchem.wikispaces.com/EXP203.

[B8] p m from compound UC-099C from EXP099 http://usefulchem.wikispaces.com/EXP099.

[B9] Bradley J-C, NMR H spectrum 203A11 from UsefulChem EXP203 http://usefulchem.wikispaces.com/EXP203.

[B10] C NMR spectrum 206A from UsefulChem EXP206 http://usefulchem.wikispaces.com/EXP206.

[B11] IR spectrum from compound UC-099C from EXP099 http://usefulchem.wikispaces.com/EXP099.

[B12] FAB-MS from compound UC-099C from EXP099 http://usefulchem.wikispaces.com/EXP099.

[B13] Analysis details available at http://usefulchem.wikispaces.com/space/showimage/EXP201-203_KO-A.xls.

[B14] Montgomery DC (2005). Design and Analysis of Experiments. Nature Protocols.

[B15] Tye H, Whittaker M (2004). Use of a Design of Experiments approach for the optimisation of a microwave assisted Ugi reaction. Org. Biomol. Chem..

